# *AMROrbit:* a trajectory-based scorecard for antimicrobial stewardship using routine susceptibility testing data

**DOI:** 10.1093/jacamr/dlag116

**Published:** 2026-07-22

**Authors:** Jasmine Kaur, Noel Abraham Tiju, Rishi Pendyala, Muthuraj Vairamuthu, Aryan Gupta, Tavpritesh Sethi

**Affiliations:** Center of Excellence in Healthcare, Indraprastha Institute of Information Technology Delhi, Okhla Industrial Estate Phase III, New Delhi 110020, India; Department of Computational Biology, Indraprastha Institute of Information Technology Delhi, Okhla Industrial Estate Phase III, New Delhi 110020, India; Center of Excellence in Healthcare, Indraprastha Institute of Information Technology Delhi, Okhla Industrial Estate Phase III, New Delhi 110020, India; Center of Excellence in Healthcare, Indraprastha Institute of Information Technology Delhi, Okhla Industrial Estate Phase III, New Delhi 110020, India; Center of Excellence in Healthcare, Indraprastha Institute of Information Technology Delhi, Okhla Industrial Estate Phase III, New Delhi 110020, India; Center of Excellence in Healthcare, Indraprastha Institute of Information Technology Delhi, Okhla Industrial Estate Phase III, New Delhi 110020, India; Center of Excellence in Healthcare, Indraprastha Institute of Information Technology Delhi, Okhla Industrial Estate Phase III, New Delhi 110020, India; Department of Computational Biology, Indraprastha Institute of Information Technology Delhi, Okhla Industrial Estate Phase III, New Delhi 110020, India

## Abstract

**Objectives:**

Antimicrobial resistance (AMR) is a serious threat to global health. However, current stewardship approaches rely on static antibiograms that overlook temporal changes in resistance. We developed *AMROrbit*, an explainable trajectory-based scorecard to proactively identify emerging AMR patterns using routinely generated antimicrobial susceptibility data.

**Methods:**

As a proof of concept, *AMROrbit* was applied to the Pfizer ATLAS dataset, including ESKAPE pathogens and *Escherichia coli* across four samples. For each antibiotic–microorganism–sample–country combination, yearly resistance percentages were modelled in rolling windows to generate estimates of resistance amplitude (baseline resistance) and velocity (rate of change). The estimates were plotted in a 2D space stratified using global medians into four quadrants. Q1 (ideal; low amplitude, low velocity), Q2 (high, low), Q3 (low, high) and Q4 (concerning; high, high). Temporal trajectories were classified as containment or spiral-in (moving towards Q1), persistent (stable within quadrant) or spiral-out (moving towards Q4).

**Results:**

Analysis revealed 62.4% of the combinations demonstrate increasing baseline resistance. Among the 5920 country-level trajectories, 17.7% depicted spiralling-out patterns, compared with only 13.4% spiralling-in, suggesting a concerning global imbalance. The patterns differed across sample-microorganism–antibiotic combinations, highlighting heterogeneity in trajectories across settings. An open-access dashboard and analytical suite are provided to generate trajectory-based stewardship insights from routine antimicrobial susceptibility testing datasets (https://amrorbit.tavlab.iiitd.edu.in:3002/).

**Conclusions:**

*AMROrbit* extends conventional surveillance by jointly characterizing resistance levels and their rate of change within an interpretable trajectory-based framework. This allows emerging resistance patterns to be identified earlier and may support more timely stewardship responses. Its reliance on routinely collected microbiology data supports scalability across diverse health system settings.

## Introduction

Antimicrobial resistance (AMR) is a global challenge, currently associated with an estimated 4.95 million deaths annually, including 1.27 million directly attributable to resistant infections.^1^ The burden is disproportionately borne by low- and middle-income countries, where infection rates are high, diagnostic capacity is constrained and overburdened health systems amplify both transmission and mortality.^2^

The global response to AMR has largely evolved around two foundational pillars, surveillance and stewardship. AMR surveillance provides systematic information on resistance patterns and trends. Antimicrobial stewardship programmes translate these data into interventions to optimize antibiotic use and improve patient outcomes.^3–5^ However, traditional stewardship approaches often depend on retrospective insights, such as annual antibiograms, which lag behind rapidly evolving resistance patterns.^6–8^ By the time such patterns are evident in aggregate summaries, opportunities for early intervention may already have been reduced.^5,7^ As a result, stewardship remains more reactive than anticipatory in most settings. Transforming retrospective data into actionable insights requires analytic frameworks capable of detecting shifts in resistance patterns early enough to inform clinical decisions.

Literature evidence shows that statistical and machine-learning approaches, coupled with dashboards and alerts, have the potential to augment traditional stewardship models by providing interpretable, near-real-time signals to frontline clinicians and policymakers when trajectories begin to emerge.^9–11^ Regression-based surveillance models have been widely used to estimate temporal trends in AMR and can support the detection of increasing or decreasing resistance over time.^12^ Dynamic regression and related time-series models extend this approach by linking antimicrobial consumption with resistance, estimating lagged associations and generating short-term forecasts where sufficiently granular antimicrobial use and resistance data are available.^13^ More recently, machine-learning approaches have used antimicrobial susceptibility testing (AST) datasets, including Pfizer ATLAS, to predict resistance phenotypes at the isolate or microorganism–antibiotic level, with emphasis on predictive performance and model interpretability.^14–16^ These approaches address important but distinct operational questions on estimating trends, identifying associations between consumption and resistance, forecasting and individual-level or isolate-level resistance prediction. However, fewer frameworks have focused on translating routine surveillance outputs into an interpretable trajectory classification that jointly captures both the current burden of resistance and its direction of change across countries, organisms, samples and antibiotics.

Our previous work in India^17–19^ has demonstrated that routinely collected surveillance data can yield actionable insights and early warning indicators and has introduced a model-based AMR scorecard for focused interventions and continuous monitoring in tertiary care settings. That framework, however, did not explicitly characterize the temporal trajectories of resistance.

To address this gap, we developed *AMROrbit*, an explainable trajectory-based scorecard that converts routine microbiology data into comparative stewardship insights. *AMROrbit* uses model-derived estimates of baseline resistance burden and temporal change to define two interpretable dimensions: amplitude and velocity. By mapping these dimensions into a shared phase space, the framework distinguishes trajectories with similar resistance trends but different baseline burdens or similar burdens but different temporal directions. *AMROrbit* is intended not as a replacement for regression, forecasting or resistance prediction models, but as a translational layer that summarizes observed AMR dynamics into trajectory states that can support surveillance interpretation, prioritization and stewardship review.

## Methods

### Data source

ATLAS is a global initiative that monitors resistance trends through standardized AST, providing longitudinal data from clinical microbiology laboratories worldwide. For this study, the PFIZER ATLAS dataset was provided through the Vivli platform as part of the AMR Surveillance Data Challenge.^20–22^ The dataset comprised longitudinal AST data (2004 onwards) from clinical microbiology laboratories worldwide, with isolates tested against a predefined panel of antimicrobials. This study included data for ESKAPE pathogens [*Enterococcus faecium*, *Staphylococcus aureus* (MRSA), *S. aureus* (MSSA)*, Klebsiella pneumoniae*, *Klebsiella aerogenes*, *Acinetobacter baumannii*, *Pseudomonas aeruginosa*, *Enterobacter cloacae* and *Enterobacter* spp.] and *Escherichia coli* from bloodstream, urine, sputum and wound infections.

To ensure sufficient temporal depth for resistance trajectory modelling, only countries with comprehensive surveillance data spanning 2014 to 2022 were included (Table S1, available as Supplementary data at *JAC-AMR* Online). The inclusion criteria for each country were assessed separately for each organism–antibiotic–sample combination. For each combination, countries were retained only if they had a complete time series across the study period. Consequently, the number and identity of included countries varied across analytical strata rather than constituting a single fixed set of countries (Table S2). Antimicrobial susceptibility results were analysed using the pre-classified susceptible, intermediate and resistant categories available in the ATLAS dataset. *AMROrbit* did not apply additional clinical breakpoints to raw MIC or zone-diameter values. Susceptible dose-dependent was not available as a separate category in the extracted data and was therefore not separately analysed. All analyses were performed on yearly aggregated data, with no patient-level identifiers used.

### Exploratory data analysis

The isolation percentage of an organism was calculated as in previous studies^17,19^:


$$\mathrm{Total}\;\mathrm{isolation}\;\mathrm{rate}\;(\mathrm{Organism}\;1)\;=\frac{\mathrm{Count}\;\mathrm{of}\;\mathrm{isolates}\;\mathrm{for}\;\mathrm{organism}\;1\;(\mathrm{nO}1)}{\mathrm{Count}\;\mathrm{of}\;\mathrm{total}\;\mathrm{positive}\;\mathrm{cultures}\;(N)}\;\times 100$$

Yearly aggregated resistance percentages were calculated for each antibiotic–organism–sample–country combination to generate a time series of resistance as


$$\mathrm{AMR}\;\mathrm{rate}\;(R\text{\%})\;=\;\frac{R\;}{R\;+I\;+S}\times 100,$$

where *R* is the count of resistant isolates, *S* is the count of susceptible isolates and *I* is the count of intermediate isolates

### *AMROrbit* scorecard

**Figure 1.**
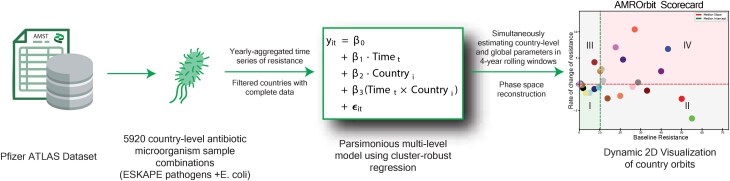
From snapshots to trajectories: *AMROrbit* pipeline and visualization of AMR dynamics. AST data for ESKAPE pathogens and *E. coli* from 4 sample types (urinary tract, bloodstream, respiratory and wound infections) were used to estimate intercept (baseline level) and slope (rate of change) of resistance using cluster-robust regression models. Estimates were derived within overlapping 4-year rolling windows (3-year overlap), allowing both global- and country-level resistance patterns to be examined over time. These estimates were then plotted in a 2D phase space based on global medians. Quadrant I represents low slope (rate of change of resistance) and low intercept (baseline resistance); Quadrant II low slope and high intercept; Quadrant III high slope and low intercept; and Quadrant IV high slope and high intercept.

Countries with complete time series (aggregated values for all years from 2014 to 2022) for the entire duration were used for modelling (Figure 1). All statistical analyses were performed in R.^23^ Data preprocessing was conducted using dplyr and lubridate. For each country–organism–sample–antibiotic combination, annual resistance percentages were calculated as the number of resistant isolates divided by the total number of isolates with valid antimicrobial susceptibility interpretations. A centred 3-year moving average was applied to the annual resistance percentage using rollmean() from the zoo package.^24^ Temporal trajectories were then estimated within rolling 4-year windows from 2014 to 2022. For each window, linear regression models were fitted using the base R lm() function from the stats package, with annual resistance percentage as the outcome and year, country and year-by-country interaction terms as predictors. From each fitted model, two parameters were extracted: (i) the intercept, representing baseline resistance (amplitude) and (ii) the slope, representing the annual rate of change (velocity). Model summaries were obtained using summary(), and coefficient tests were generated using coeftest() from the lmtest package^25^ with robust covariance estimates from the vcovHAC() in the sandwich package.^26^ The *AMROrbit* scorecard visualization was performed in Python using pandas, numpy and matplotlib.^27–29^ Regression-derived amplitude and velocity estimates were mapped into a 2D phase space and stratified using global medians into four quadrants: Q1 (ideal): low baseline resistance and low rate of change; Q2: high baseline resistance and low rate of change; Q3: low baseline resistance and high rate of change; Q4 (concerning): high baseline resistance and high rate of change.

Country-level trajectories were classified by comparing the first and final rolling-window quadrants. Spiral-in (containment) was defined as movement from Q2, Q3 or Q4 to Q1; spiral-out (emerging risk) as movement from Q1, Q2 or Q3 to Q4; persistent as remaining in the same quadrant; and mixed as all other quadrant transitions.

The global median thresholds were not used for statistical estimation itself, but rather for the interpretive phase-space representation layered on top of the continuous regression-derived parameters. The underlying model retained resistance burden and temporal change as continuous quantities, while the quadrant-based scorecard provided a pragmatic stewardship abstraction for communicating dynamic AMR states.

To account for the non-independence of trajectory observations from the same country across antibiotics, we used a country-level cluster bootstrap (10 000 replicates). In each replicate, countries were resampled with replacement within each organism–source stratum, and all antibiotic-specific trajectory records for each sampled country were retained. The 2.5th and 97.5th percentiles of the bootstrap distribution were taken as the 95% CI for the proportion of country–antibiotic observations in each trajectory category.

### Sensitivity analysis for the global median threshold

To assess sensitivity to the choice of phase-space thresholds, we repeated the trajectory classification using region-specific medians instead of global medians. Countries were grouped into four geographic regions: Africa and Middle East, the Americas, Asia and Europe (Table S3). For each organism–sample–antibiotic combination, region-specific medians for baseline resistance and velocity were estimated and used to define the corresponding phase-space quadrants. Regional median–based trajectory classifications were then compared with the original global median–based classifications across all country-level trajectories.

### Web-based tool for scorecard

To support application of the *AMROrbit* scorecard to local datasets, we developed a web-based interface that allows users to upload routine microbiology data, map antimicrobial susceptibility results to standardized categories and generate trajectory-based scorecards. The interface incorporates basic data validation checks and provides interactive visualizations of resistance trends and trajectories.

## Results

Across the full ATLAS dataset, 5920 country-level antibiotic–microorganism–sample combinations (212 434 records) were analysed from 2014 to 2022. Aggregated global results revealed 62.4% of combinations showed rising baseline resistance, indicating a broad upward shift in AMR burden over time. Among country-level combinations, 17.7% were spiralling-out compared with only 13.4% spiralling-in, revealing a concerning global imbalance that presents opportunities for policy learning and adaptation. A majority of the combinations followed persistent/mixed resistance trajectories, suggesting sustained resistance levels without substantial improvement or deterioration (Table 1).

**Table 1.** Summary of country–organism–source–antibiotic AMROrbit trajectories by organism and sample source

Organism	Source	Spiralling-in	Spiralling-out	Persistent	Other/mixed
*Acinetobacter baumannii*	Blood	23/96 (24.0%; 95% CI 7.3–44.8)	21/96 (21.9%; 95% CI 7.3–39.6)	34/96 (35.4%; 95% CI 22.9–47.9)	18/96 (18.8%; 95% CI 7.3–31.3)
*Acinetobacter baumannii*	Sputum	26/85 (30.6%; 95% CI 9.2–54.8)	20/85 (23.5%; 95% CI 8.3–40.5)	24/85 (28.2%; 95% CI 9.5–47.7)	15/85 (17.6%; 95% CI 4.7–34.1)
*Acinetobacter baumannii*	Urine	22/84 (26.2%; 95% CI 8.3–47.6)	19/84 (22.6%; 95% CI 4.8–44.0)	24/84 (28.6%; 95% CI 11.9–47.6)	19/84 (22.6%; 95% CI 7.1–40.5)
*Acinetobacter baumannii*	Wound	8/72 (11.1%; 95% CI 0.0–25.0)	8/72 (11.1%; 95% CI 0.0–29.2)	27/72 (37.5%; 95% CI 20.8–55.6)	29/72 (40.3%; 95% CI 22.2–59.7)
*Enterobacter cloacae*	Blood	24/181 (13.3%; 95% CI 8.1–18.9)	27/181 (14.9%; 95% CI 9.6–20.7)	38/181 (21.0%; 95% CI 16.9–25.0)	92/181 (50.8%; 95% CI 45.9–56.0)
*Enterobacter cloacae*	Sputum	18/145 (12.4%; 95% CI 5.9–19.0)	35/145 (24.1%; 95% CI 16.2–31.8)	31/145 (21.4%; 95% CI 13.9–28.8)	61/145 (42.1%; 95% CI 32.7–53.3)
*Enterobacter cloacae*	Urine	23/153 (15.0%; 95% CI 6.9–23.9)	34/153 (22.2%; 95% CI 15.0–29.0)	37/153 (24.2%; 95% CI 17.6–29.5)	59/153 (38.6%; 95% CI 31.0–49.1)
*Enterobacter cloacae*	Wound	16/164 (9.8%; 95% CI 4.4–15.3)	30/164 (18.3%; 95% CI 12.1–25.4)	42/164 (25.6%; 95% CI 18.7–32.4)	76/164 (46.3%; 95% CI 38.8–55.3)
*Enterobacter* species^a^	Blood	4/44 (9.1%; 95% CI 2.4–16.0)	2/44 (4.5%; 95% CI 0.0–14.3)	9/44 (20.5%; 95% CI 8.8–28.3)	29/44 (65.9%; 95% CI 54.3–83.3)
*Enterobacter* species^a^	Sputum	2/35 (5.7%; 95% CI 0.0–9.7)	6/35 (17.1%; 95% CI 0.0–29.0)	10/35 (28.6%; 95% CI 5.3–42.9)	17/35 (48.6%; 95% CI 27.5–73.7)
*Enterobacter* species^a^	Urine	0/11 (0.0%; 95% CI 0.0–0.0)	0/11 (0.0%; 95% CI 0.0–0.0)	0/11 (0.0%; 95% CI 0.0–0.0)	11/11 (100.0%; 95% CI 100.0–100.0)
*Enterobacter* species^a^	Wound	3/23 (13.0%; 95% CI 0.0–28.1)	2/23 (8.7%; 95% CI 0.0–18.8)	1/23 (4.3%; 95% CI 0.0–9.4)	17/23 (73.9%; 95% CI 70.7–100.0)
*Enterococcus faecium*	Blood	6/80 (7.5%; 95% CI 2.4–13.3)	9/80 (11.2%; 95% CI 3.8–20.3)	20/80 (25.0%; 95% CI 15.2–34.1)	45/80 (56.2%; 95% CI 48.8–64.2)
*Enterococcus faecium*	Sputum	0/1 (0.0%; 95% CI 0.0–0.0)	0/1 (0.0%; 95% CI 0.0–0.0)	0/1 (0.0%; 95% CI 0.0–0.0)	1/1 (100.0%; 95% CI 100.0–100.0)
*Enterococcus faecium*	Urine	4/56 (7.1%; 95% CI 0.0–16.7)	8/56 (14.3%; 95% CI 3.8–26.7)	20/56 (35.7%; 95% CI 25.0–46.4)	24/56 (42.9%; 95% CI 35.0–51.8)
*Enterococcus faecium*	Wound	3/30 (10.0%; 95% CI 0.0–23.1)	5/30 (16.7%; 95% CI 0.0–29.4)	10/30 (33.3%; 95% CI 13.6–52.9)	12/30 (40.0%; 95% CI 28.9–61.1)
*Escherichia coli*	Blood	47/333 (14.1%; 95% CI 9.6–18.9)	60/333 (18.0%; 95% CI 12.5–24.1)	102/333 (30.6%; 95% CI 24.5–36.7)	124/333 (37.2%; 95% CI 30.5–44.4)
*Escherichia coli*	Sputum	29/218 (13.3%; 95% CI 8.2–18.7)	42/218 (19.3%; 95% CI 11.9–27.7)	79/218 (36.2%; 95% CI 30.6–41.8)	68/218 (31.2%; 95% CI 24.7–38.2)
*Escherichia coli*	Urine	52/297 (17.5%; 95% CI 11.4–23.9)	51/297 (17.2%; 95% CI 12.2–22.9)	105/297 (35.4%; 95% CI 29.5–41.6)	89/297 (30.0%; 95% CI 24.2–36.4)
*Escherichia coli*	Wound	37/258 (14.3%; 95% CI 8.9–20.2)	54/258 (20.9%; 95% CI 14.0–28.5)	76/258 (29.5%; 95% CI 23.2–35.9)	91/258 (35.3%; 95% CI 27.5–43.4)
*Klebsiella aerogenes*	Blood	12/103 (11.7%; 95% CI 3.8–21.6)	10/103 (9.7%; 95% CI 3.4–16.7)	21/103 (20.4%; 95% CI 11.7–29.5)	60/103 (58.3%; 95% CI 51.4–68.0)
*Klebsiella aerogenes*	Sputum	7/94 (7.4%; 95% CI 2.1–12.9)	18/94 (19.1%; 95% CI 12.0–25.7)	27/94 (28.7%; 95% CI 23.3–34.2)	42/94 (44.7%; 95% CI 36.9–55.4)
*Klebsiella aerogenes*	Urine	8/94 (8.5%; 95% CI 2.6–14.8)	22/94 (23.4%; 95% CI 13.0–32.7)	24/94 (25.5%; 95% CI 18.8–31.1)	40/94 (42.6%; 95% CI 34.3–54.4)
*Klebsiella aerogenes*	Wound	4/54 (7.4%; 95% CI 0.0–16.4)	8/54 (14.8%; 95% CI 6.7–20.0)	7/54 (13.0%; 95% CI 3.8–20.0)	35/54 (64.8%; 95% CI 53.8–85.2)
*Klebsiella pneumoniae*	Blood	58/324 (17.9%; 95% CI 12.0–24.4)	64/324 (19.8%; 95% CI 11.4–29.3)	115/324 (35.5%; 95% CI 29.6–41.7)	87/324 (26.9%; 95% CI 20.7–33.3)
*Klebsiella pneumoniae*	Sputum	47/361 (13.0%; 95% CI 7.9–18.8)	70/361 (19.4%; 95% CI 12.9–26.6)	122/361 (33.8%; 95% CI 26.6–40.9)	122/361 (33.8%; 95% CI 27.1–40.9)
*Klebsiella pneumoniae*	Urine	48/325 (14.8%; 95% CI 8.8–21.4)	67/325 (20.6%; 95% CI 11.7–30.4)	98/325 (30.2%; 95% CI 24.2–36.2)	112/325 (34.5%; 95% CI 26.4–42.9)
*Klebsiella pneumoniae*	Wound	38/317 (12.0%; 95% CI 7.3–17.2)	46/317 (14.5%; 95% CI 8.4–22.0)	109/317 (34.4%; 95% CI 27.4–41.5)	124/317 (39.1%; 95% CI 31.9–46.5)
*Pseudomonas aeruginosa*	Blood	30/203 (14.8%; 95% CI 7.9–22.4)	32/203 (15.8%; 95% CI 8.0–24.9)	64/203 (31.5%; 95% CI 23.5–39.7)	77/203 (37.9%; 95% CI 29.8–47.0)
*Pseudomonas aeruginosa*	Sputum	43/330 (13.0%; 95% CI 8.0–18.6)	72/330 (21.8%; 95% CI 13.5–30.9)	105/330 (31.8%; 95% CI 25.5–38.3)	110/330 (33.3%; 95% CI 24.6–42.7)
*Pseudomonas aeruginosa*	Urine	40/288 (13.9%; 95% CI 8.2–20.2)	54/288 (18.8%; 95% CI 11.1–27.5)	94/288 (32.6%; 95% CI 25.3–40.1)	100/288 (34.7%; 95% CI 25.7–44.6)
*Pseudomonas aeruginosa*	Wound	52/255 (20.4%; 95% CI 12.3–29.0)	49/255 (19.2%; 95% CI 10.3–29.1)	82/255 (32.2%; 95% CI 24.9–39.8)	72/255 (28.2%; 95% CI 20.3–36.7)
*Staphylococcus aureus* (MRSA)	Blood	4/25 (16.0%; 95% CI 4.0–32.0)	4/25 (16.0%; 95% CI 4.0–32.0)	10/25 (40.0%; 95% CI 20.0–60.0)	7/25 (28.0%; 95% CI 12.0–48.0)
*Staphylococcus aureus* (MRSA)	Sputum	6/109 (5.5%; 95% CI 1.5–9.6)	9/109 (8.3%; 95% CI 2.8–14.1)	24/109 (22.0%; 95% CI 14.8–28.1)	70/109 (64.2%; 95% CI 57.0–73.6)
*Staphylococcus aureus* (MRSA)	Urine	1/34 (2.9%; 95% CI 0.0–8.6)	1/34 (2.9%; 95% CI 0.0–8.5)	5/34 (14.7%; 95% CI 0.0–28.3)	27/34 (79.4%; 95% CI 64.4–100.0)
*Staphylococcus aureus* (MRSA)	Wound	11/188 (5.9%; 95% CI 2.7–9.2)	31/188 (16.5%; 95% CI 12.6–20.4)	66/188 (35.1%; 95% CI 28.9–41.3)	80/188 (42.6%; 95% CI 35.9–50.0)
*Staphylococcus aureus* (MSSA)	Blood	7/66 (10.6%; 95% CI 4.5–18.2)	5/66 (7.6%; 95% CI 1.5–13.6)	34/66 (51.5%; 95% CI 39.4–63.6)	20/66 (30.3%; 95% CI 19.7–42.4)
*Staphylococcus aureus* (MSSA)	Sputum	12/141 (8.5%; 95% CI 3.9–13.2)	25/141 (17.7%; 95% CI 12.1–24.0)	35/141 (24.8%; 95% CI 19.0–30.5)	69/141 (48.9%; 95% CI 44.4–54.1)
*Staphylococcus aureus* (MSSA)	Urine	6/65 (9.2%; 95% CI 2.9–16.7)	15/65 (23.1%; 95% CI 12.5–33.3)	12/65 (18.5%; 95% CI 8.3–29.0)	32/65 (49.2%; 95% CI 40.3–60.7)
*Staphylococcus aureus* (MSSA)	Wound	12/178 (6.7%; 95% CI 2.8–11.4)	13/178 (7.3%; 95% CI 3.3–11.9)	58/178 (32.6%; 95% CI 27.2–37.7)	95/178 (53.4%; 95% CI 48.9–58.3)

Percentages are calculated using the total number of country–antibiotic trajectory observations within each organism–sample stratum as the denominator. Spiralling-in is defined as movement from QII, QIII or QIV at baseline to QI in the final time window; spiralling-out is defined as movement from QI, QII or QIII at baseline to QIV in the final time window; persistent trajectories are defined as trajectories remaining in the same quadrant; and other/mixed trajectories included non-directional, oscillatory or mixed quadrant sequences that did not meet the predefined criteria for spiralling-in, spiralling-out or persistent classification. Ninety-five percent CIs for trajectory proportions were estimated using a country-level cluster bootstrap with 10 000 replicates. In each replicate, countries were resampled with replacement within each organism–sample stratum, retaining all antibiotic-specific trajectory records for the sampled countries, and the 2.5th and 97.5th percentiles of the bootstrap distribution were used as the confidence limits. Values represent the number and percentage of country-level trajectories assigned to each category. Trajectory categories were derived from post-estimation movement across *AMROrbit* quadrants using predefined rules and were not tested as separate inferential outcomes; therefore, *P*-values are not reported for these classifications.

^a^ *Enterobacter* species includes *Enterobacter asburiae*, *Enterobacter kobei*, *Enterobacter ludwigii*, *Enterobacter cancerogenus*, *Enterobacter gergoviae*, and *Enterobacter* spp.

### Variation across microorganism–sample combinations

Table 1 summarizes *AMROrbit* trajectory classifications at the level of country–organism–sample–antibiotic trajectories. Table S1 provides the corresponding country- and antibiotic-level trajectory output, with the trajectory classification for each organism–sample–antibiotic combination. A higher proportion of spiral-out trajectories was observed in respiratory isolates for *En. cloacae* (24.1%), *A. baumannii* (23.5%), *P. aeruginosa* (21.8%) and urinary isolates for *Staphylococcus* (MSSA; 23.1%) and *K. aerogenes* (23.4%; Table 1).

To evaluate the robustness of the scorecard to threshold selection, we performed a sensitivity analysis using region-specific median thresholds as alternative decision boundaries. We defined directional discordance as a direct reversal between the two most stewardship-relevant directional categories: spiralling-in under one thresholding approach and spiralling-out under the other or vice versa. Reclassifications involving constant or mixed categories were not treated as directional reversals but were considered changes in exact category assignment.

Using this definition, 5890 of 5920 trajectories showed directional concordance, corresponding to 99.49%. Direct reversal was uncommon. Only 12 trajectories changed from spiralling-in under global thresholds to spiralling-out under regional thresholds (0.20%), while 18 trajectories changed from spiralling-out under global thresholds to spiralling-in under regional thresholds (0.30%). Overall, 30 trajectories were directionally discordant, accounting for 0.51% of all trajectories. These findings indicate that the principal directional interpretation of *AMROrbit* was largely robust to the use of global versus region-specific threshold boundaries, despite some exact category reclassification.

### Early-warning insights

**Figure 2.**
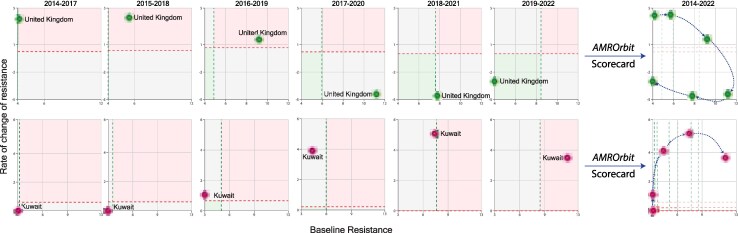
*AMROrbit* scorecard. The *x* and *y* axes represent intercept (baseline resistance) and slope (rate of change). Country trajectories are classified into intuitive patterns: spiralling-in (positive AMR containment), persistent resistance (remaining in the same quadrant) and spiralling-out (negative AMR trajectory). The UK demonstrates a positive trajectory, while Kuwait’s trajectory signals deterioration, shifting from Quadrants I to IV, indicating an urgent need for targeted intervention.

Tracking trajectories through *AMROrbit* can enable identification of directional deterioration in resistance velocity before large changes in absolute resistance burden are apparent, with the achievable time scale of detection determined by the temporal granularity and density of the underlying surveillance data. For example, in Kuwait’s *K. pneumoniae*–Amikacin–blood trajectory, Kuwait remained close to global median levels during the 2014–17 window, with a stable or declining slope. However, in the subsequent 2015–18 window, an increase in slope was observed, preceding a transition into a spiral-out trajectory and, later, into the high-risk quadrant (Q4). This example illustrates how changes in trajectory (velocity) can precede increases in baseline resistance (amplitude), providing an earlier signal of deterioration than static summaries. Figure 2 depicts the UK following a spiral-in trajectory consistent with improving resistance dynamics, whereas Kuwait exhibits a spiral-out trajectory indicating progressive deterioration.

### Open-source web-based interface for trajectory-based AMR analysis

**Figure 3.**
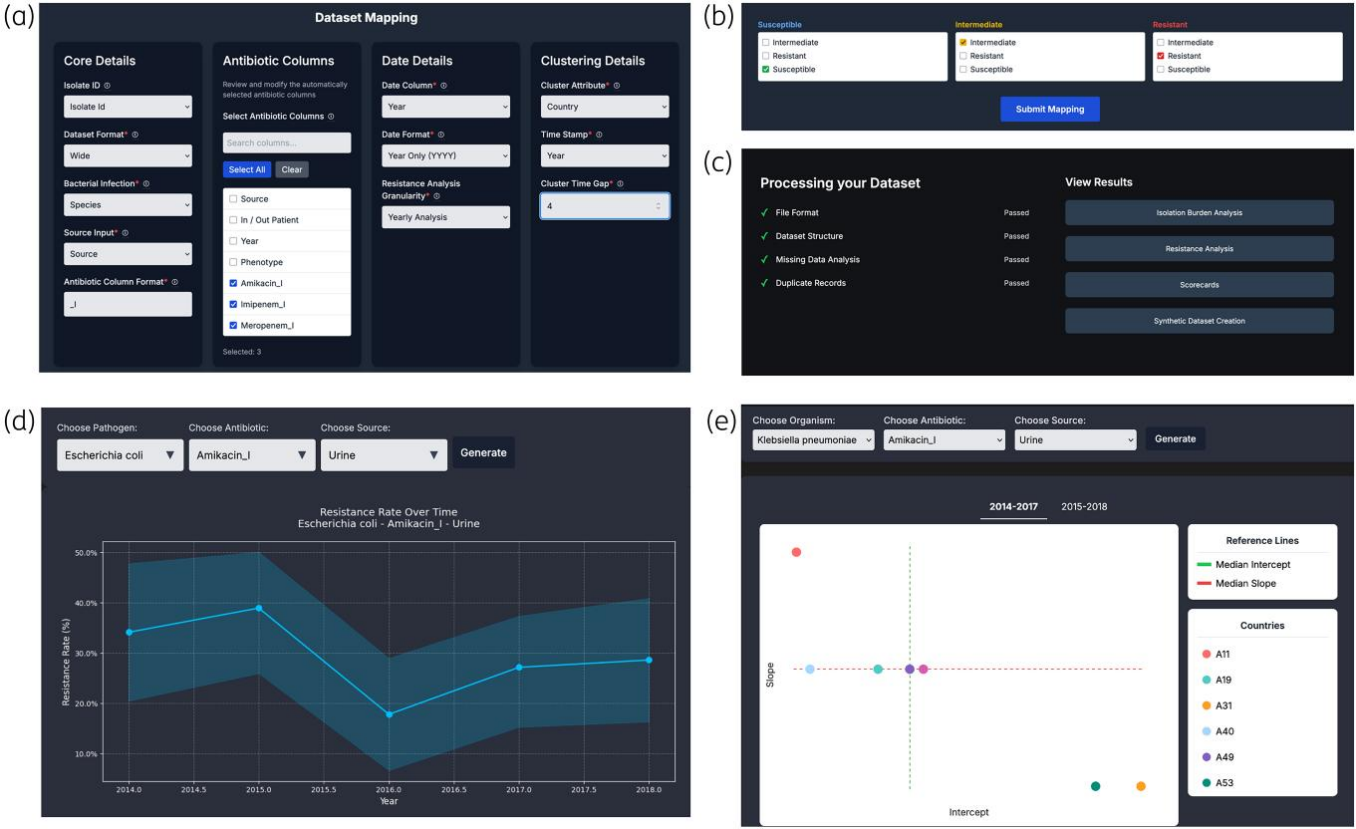
Workflow for applying the *AMROrbit* scorecard to routine microbiology data. Screenshots showing the main steps in the analysis: (a) dataset and variable mapping; (b) conversion of AST results into susceptible, intermediate and resistant categories; (c) basic data checks and analysis, including scorecard generation; (d) visualization of longitudinal resistance trends for selected organism–antibiotic–sample combinations and (e) the trajectory-based *AMROrbit* scorecard, showing baseline resistance and rate of change relative to reference medians.

To support the application of the *AMROrbit* scorecard across different settings, we developed a web-based interface that allows analysis of routinely collected microbiology data (Figure 3). The platform enables harmonization of the dataset, standardization of AST outputs and automated generation of trajectory-based scorecards. Users can explore longitudinal resistance patterns and associated trajectories for selected microorganism–antibiotic–sample combinations, facilitating consistent interpretation of resistance dynamics across datasets.

## Discussion

This study developed and demonstrated *AMROrbit*, a trajectory-based scorecard for translating routinely generated AST data into interpretable summaries of AMR dynamics. Conventional AMR outputs often emphasize either resistance prevalence at a given point or temporal trends estimated separately. While both are valuable, neither alone is sufficient for stewardship prioritization. A high-resistance combination that is stable over time may require a different programmatic response from a lower-resistance combination that is worsening rapidly. *AMROrbit* addresses this interpretive gap by jointly representing baseline resistance burden and temporal velocity within a shared phase-space structure.

Applied to 5920 country-level antibiotic–microorganism–sample trajectories from the ATLAS dataset, *AMROrbit* showed that AMR trajectories were heterogeneous across countries, organisms, samples and antibiotics. Importantly, baseline resistance burden and temporal change did not always provide the same signal, while some combinations had high-resistance levels but relatively stable trends, others had lower baseline resistance but increasing velocity. These findings suggest that *AMROrbit* can move beyond static descriptions of resistance burden and provide a compact framework for comparative AMR surveillance.

*AMROrbit* should be viewed as complementary to the existing regression-based, dynamic time-series and machine-learning approaches in AMR surveillance. While regression and time-series models remain essential for estimating temporal trends, examining antimicrobial consumption–resistance relationships and supporting forecasts where sufficiently granular data are available. *AMROrbit* adds an interpretive layer that converts continuous estimates of resistance burden and temporal change into trajectory classes that can be compared across settings. This is operationally relevant since the same slope may have different implications depending on the baseline resistance state from which it emerges.

The ATLAS application illustrates this distinction. Antibiotic–microorganism–sample combinations classified as spiralling-out may identify settings with high, increasing or both high and increasing resistance, warranting closer stewardship review. Persistent high-resistance trajectories may indicate combinations in which existing stewardship measures need to be maintained or strengthened, while spiralling-in trajectories may help identify contexts where resistance is moving in a favourable direction and where local practices warrant further examination. Low-amplitude but increasing-velocity trajectories may also be useful as early-warning signals, as they can identify emerging increases in resistance that may not yet be evident from prevalence-based thresholds alone. From a stewardship perspective, these classifications are best interpreted as prioritization signals rather than prescriptive treatment rules.

The thresholding strategy is an interpretive choice. Global medians were used in the primary analysis to provide a common reference frame for cross-country comparison. However, global thresholds may not fully reflect regional epidemiology, surveillance practices or stewardship priorities. The regional-median sensitivity analysis showed that some classifications changed when countries were compared within a more local comparator distribution, although direct reversal between spiralling-in and spiralling-out trajectories was uncommon. This suggests that the main directional signal was generally stable, but that *AMROrbit* labels should be interpreted in relation to the thresholding frame used. Global thresholds may be useful for international benchmarking, whereas regional or local thresholds may be preferable for programme-level stewardship decisions.

Our approach has some limitations. First, the present ATLAS-based analysis was conducted at annual granularity. Because each antibiotic–microorganism–sample–country series contained a limited number of yearly observations, we did not impute missing annual values. Instead, *AMROrbit* was applied to combinations with complete longitudinal data to avoid introducing model-dependent artefacts into the estimation of resistance trajectories. This conservative approach supports consistent comparison across included combinations but may under-represent countries, organisms, samples or antibiotics with sparse or intermittent reporting. Second, the ATLAS dataset provides a large, standardized, multi-country AMR dataset suitable for demonstrating the *AMROrbit* scorecard, but the resulting trajectories should not be interpreted as fully nationally representative estimates for every included country. Resistance patterns may reflect participating surveillance sites, submitted isolates, patient populations, sample distribution, diagnostic practices and antibiotic testing panels within ATLAS. Therefore, *AMROrbit* classifications derived from this dataset should be interpreted as dynamic patterns among analysable country-level series in ATLAS, rather than as definitive national resistance rankings. Third, although *AMROrbit* was demonstrated here using annual ATLAS data, the scorecard is adaptable to datasets with finer temporal granularity and different geographic levels. In hospital, regional or national surveillance systems with more granular AST data, missingness could be addressed using pre-specified imputation, smoothing or sensitivity-analysis approaches before trajectory estimation, provided these choices are appropriate to the data structure, missingness mechanism and reporting process. The generalizability of *AMROrbit’s* outputs will therefore depend not only on the scorecard itself but also on local data quality, sampling structure, microbiology practices, breakpoint standards, temporal resolution and stewardship context. Prospective implementation in locally governed surveillance systems will be needed to assess whether the classifications are stable, actionable and useful for stewardship review. We are currently implementing *AMROrbit* in two tertiary care hospitals in New Delhi, which will allow future assessment of its feasibility, interpretability and operational utility in local stewardship workflows. A further limitation is that resistance-mechanism–specific annotation was not consistently available in ATLAS. For example, although *En. cloacae* complex is highly relevant for chromosomal AmpC-mediated resistance, only a small subset of isolates had known AmpC data (Table S4 available as Supplemental data on JAC-AMR online). This precluded reliable temporal modelling of AmpC-specific trajectories. *AMROrbit* could be extended to such mechanism-specific surveillance questions where phenotypic or genomic resistance annotations are sufficiently complete over time.

### Conclusions

*AMROrbit* provides an interpretable scorecard for summarizing AMR trajectories using routinely generated AST data. By jointly representing baseline resistance burden and temporal velocity, the scorecard can help distinguish combinations that are persistently high, improving or moving towards higher-risk states. In the ATLAS demonstration, *AMROrbit* provided a scalable way to visualize and compare dynamic resistance patterns across country-level antibiotic–microorganism–sample combinations. The scorecard should be interpreted as a hypothesis-generating and prioritization tool, not as a replacement for local antibiograms, clinical judgement or formal stewardship decision-making. Further validation using more granular local surveillance data and prospective implementation studies will be needed to determine whether *AMROrbit* improves stewardship review, prioritization or intervention planning in real-world settings.

## Supplementary Material

dlag116_Supplementary_Data

## Data Availability

This study used Pfizer ATLAS data accessed through the Vivli AMR Register as part of the 2024 Vivli AMR Surveillance Data Challenge. Researchers may request access directly through the Vivli AMR Register, subject to Vivli/Pfizer approval and data-use terms.

## Code availability

All custom scripts and code for the *AMROrbit* scorecard presented in this study are publicly available on the GitHub repository at https://github.com/tavlab-iiitd/AMROrbit. The real-time dashboard and a web-based framework to build custom scorecards are available at https://amrorbit.tavlab.iiitd.edu.in:3002.

## Supplementary data

Tables S1 to S4 are available as Supplementary data at *JAC-AMR* Online.
